# Supplementary material for: Complexome profiling of the Chlamydomonas psb28 mutant reveals TEF5 as an early PSII assembly factor
Source: Plant Cell. 2025 Mar 17;37(6):koaf055. doi: 10.1093/plcell/koaf055 (PMC12164590; doi:10.1093/plcell/koaf055)
Supplement: koaf055_Supplementary_Data [file koaf055_supplementary_data.zip › psb28_interactive.zip!/plots/intensity/qualityPlots/peptides/_Cre12.g512000.t1.1.html]

### Cre12.g512000.t1.1

Trivial name:   
SeqCoverage [median]: 18.30  
Mass: 120.20 kDa  
  
 

| Sequence | MissedCleavage | Mass [Da] | wtIntensity | muIntensity | Score |
| --- | --- | --- | --- | --- | --- |
| AAAAAAEAVNAAVEAAADEQQQQEETTDKK | 1 | 3028.42 | 2197.09 | 30643.28 | 156.58 |
| AAAAAAEAVNAAVEAAADEQQQQEETTDKKATK | 2 | 3328.60 | 697.70 | 3788.68 | 59.05 |
| AADAKPATETKPAATETKPADTK | 3 | 2312.19 | 0.00 | 1097.69 | 49.50 |
| AADAQPADTK | 0 | 986.47 | 6105.32 | 13544.95 | 156.35 |
| ATLFDEEVR | 0 | 1078.53 | 0.00 | 1203.11 | 62.00 |
| DANAGSAAIAK | 0 | 987.50 | 7315.91 | 16458.65 | 121.73 |
| DIEPGNAR | 0 | 870.42 | 0.00 | 1024.49 | 68.92 |
| EVYYYEK | 0 | 992.45 | 0.00 | 1416.98 | 77.74 |
| FRGDSDRDYTLVVK | 2 | 1669.84 | 361.91 | 12726.09 | 71.24 |
| FSENIDFQYPMK | 0 | 1517.69 | 0.00 | 4544.07 | 205.13 |
| GDSDRDYTLVVK | 1 | 1366.67 | 0.00 | 1407.17 | 65.18 |
| GYELEMSK | 0 | 955.43 | 0.00 | 726.02 | 82.67 |
| IEITTGIPTVVR | 0 | 1297.76 | 0.00 | 7721.28 | 146.11 |
| KDANAGSAAIAK | 1 | 1115.59 | 1051.27 | 916.33 | 134.18 |
| KYLQLAAQDIR | 1 | 1317.74 | 0.00 | 1897.77 | 94.36 |
| LADIAEPPNMK | 0 | 1197.61 | 3813.13 | 20874.32 | 142.55 |
| NSNINANVPLAK | 0 | 1253.67 | 5389.86 | 22134.18 | 217.14 |
| QEMENADDIR | 0 | 1219.51 | 0.00 | 918.75 | 105.20 |
| YLQLAAQDIR | 0 | 1189.65 | 1292.33 | 19161.16 | 118.18 |
| YQVINK | 0 | 763.42 | 1495.07 | 9571.44 | 122.51 |
|  |  |  | 29719.6 | 171776.4 |  |
